# Impact of patient characteristics on innate immune responses and inflammasome activation in *ex vivo* human lung tissues infected with influenza A virus

**DOI:** 10.3389/fcimb.2023.1269329

**Published:** 2023-10-13

**Authors:** Chung-Guei Huang, Yi-Cheng Wu, Ming-Ju Hsieh, Ya-Jhu Lin, Tzu-Hsuan Hsieh, Po-Wei Huang, Shu-Li Yang, Kuo-Chien Tsao, Shin-Ru Shih, Li-Ang Lee

**Affiliations:** ^1^ Department of Laboratory Medicine, Chang Gung Memorial Hospital, Linkou Main Branch, Taoyuan, Taiwan; ^2^ Department of Medical Biotechnology and Laboratory Science, Chang Gung University, Taoyuan, Taiwan; ^3^ Research Center for Emerging Viral Infections, Chang Gung University, Taoyuan, Taiwan; ^4^ Division of Thoracic and Cardiovascular Surgery, Department of Surgery, Chang Gung Memorial Hospital, Linkou Main Branch, Taoyuan, Taiwan; ^5^ Faculty of Medicine, College of Medicine, Chang Gung University, Taoyuan, Taiwan; ^6^ School of Medicine, College of Life Sciences and Medicine, National Tsing Hua University, Hsinchu, Taiwan; ^7^ Department of Otorhinolaryngology - Head and Neck Surgery, Chang Gung Memorial Hospital, Linkou Main Branch, Taoyuan, Taiwan

**Keywords:** influenza A virus, *ex vivo* infection, cytokine, programmed death 1/programmed death-ligand 1, toll-like receptor

## Abstract

**Background:**

Influenza A virus (IAV) infection poses a persistent global health challenge, necessitating a nuanced grasp of host immune responses for optimal interventions. While the interplay between aging, immunosenescence, and IAV is recognized as key in severe lower respiratory tract infections, the role of specific patient attributes in shaping innate immune reactions and inflammasome activity during IAV infection remains under-investigated. In this study, we utilized an *ex vivo* infection model of human lung tissues with H3N2 IAV to discern relationships among patient demographics, IAV nucleoprotein (NP) expression, toll-like receptor (TLR) profiles, PD-1/PD-L1 markers, and cytokine production.

**Methods:**

Our cohort consisted of thirty adult patients who underwent video-assisted thoracoscopic surgery during 2018–2019. Post-surgical lung tissues were exposed to H3N2 IAV for *ex vivo* infections, and the ensuing immune responses were profiled using flow cytometry.

**Results:**

We observed pronounced IAV activity within lung cells, as indicated by marked NP upregulation in both epithelial cells (*P* = 0.022) and macrophages (*P* = 0.003) in the IAV-exposed group relative to controls. Notably, interleukin-2 levels correlated with variations in TLR1 expression on epithelial cells and PD-L1 markers on macrophages. Age emerged as a modulating factor, dampening innate immune reactions, as evidenced by reduced interleukin-2 and interferon-γ concentrations (both adjusted *P* < 0.05). Intriguingly, a subset of participants with pronounced tumor necrosis factor-alpha post-mock infection (Cluster 1) showed attenuated cytokine responses in contrast to their counterparts in Cluster 2 and Cluster 3 (all adjusted *P* < 0.05). Individuals in Cluster 2, characterized by a low post-mock infection NP expression in macrophages, exhibited reduced variations in both NP and TLR1–3 expressions on these cells and a decreased variation in interleukin-2 secretion in comparison to their Cluster 3 counterparts, who were identified by their elevated NP macrophage expression (all adjusted *P* < 0.05).

**Conclusion:**

Our work elucidates the multifaceted interplay of patient factors, innate immunity, and inflammasome responses in lung tissues subjected to *ex vivo* H3N2 IAV exposure, reflecting real-world lower respiratory tract infections. While these findings provide a foundation for tailored therapeutic strategies, supplementary studies are requisite for thorough validation and refinement.

## 1 Introduction

Lower respiratory tract infections, which include pneumonia, influenza, bronchitis, and bronchiolitis, are a significant global health concern and rank as the fifth leading cause of death worldwide (Troeger et al., 2017). Among these, influenza notably contributes to the burden of lower respiratory tract infections (Feldman and Shaddock, 2019). Historical data from the United States shows that, between the 1979-1980 and 2000-2001 seasons, H3N2 influenza A virus (IAV) led to the most hospitalizations and deaths (Thompson et al., 2003; Thompson et al., 2004). Notably, in Turkey’s 2015-2016 season, the fatality rate of H3N2 IAV exceeded that of H1N1 IAV (Tekin et al., 2019), emphasizing the significant threat of the H3N2 strain.

Various risk factors heighten the susceptibility to acute or severe lower respiratory tract infections. These risks encompass aging, existing health conditions, weakened immunity, smoking, incomplete vaccination, and socio-environmental factors such as overcrowding and malnutrition (Thompson et al., 2004; Lozada-Requena and Nunez Ponce, 2020). Aging particularly impacts both innate and adaptive immunity, primarily through toll-like receptors (TLRs) and the programmed death 1 (PD-1)/programmed death-ligand 1 (PD-L1) pathways (Lages et al., 2010; Shaw et al., 2011; Schneider et al., 2021). TLRs are essential in identifying pathogen-associated and damage-associated molecular patterns, empowering innate immune cells to counteract microbial invasions and activate the inflammasome (O'Neill et al., 2013; Hoque et al., 2014). Previous research has identified a notable decrease in TLR agonist response after the age of 65 (Zareian et al., 2019). Conversely, the PD-1/PD-L1 pathway, an inhibitory mechanism, preserves immune response equilibrium and immunotolerance. However, its excessive activation during cancer or viral infections can suppress T cell activity and inflammasome activation (Ai et al., 2020; Zhang Q. et al., 2020; Duhalde Vega et al., 2022). Modulating the PD-1/PD-L1 pathway offers potential therapeutic interventions for viral infections and immune-related disorders (Chamoto et al., 2017).

Furthermore, the age-associated decline in immune function, known as immunosenescence, augments vulnerability to infections (Rodrigues et al., 2021). As an example, our prior research identified differences in H7N9 IAV replication between younger (21-64 years) and older (≥ 65 years) individuals (Huang et al., 2018). Besides aging, comorbidities like lung cancer (Yu et al., 2016) and hypertension (Nunes et al., 2019) potentially influence TLRs and the PD-1/PD-L1 pathways, further contributing to immunosenescence (Ferrara et al., 2017). This compromised immune function makes individuals more susceptible to viral and bacterial infections. Yet, the comprehensive influence of patient-specific factors on H3N2 IAV infection remains relatively unexplored.

In this study, we set out to: (1) assess the effects of H3N2 IAV on primary lung tissue cultures, emphasizing TLRs, the PD-1/PD-L1 pathway, and other immune biomarkers, and (2) determine the role of patient characteristics, especially age, in H3N2 IAV infection using an *ex vivo* lung tissue model. We theorized that specific patient traits might impact the immune response during the early stages of *ex vivo* H3N2 IAV infection. Our exploration aims to clarify the intricate relationships between patient characteristics, immune responses, and H3N2 IAV infection, potentially guiding personalized influenza treatment strategies.

## 2 Materials and methods

### 2.1 Study participants

The study protocol was approved by the Institutional Review Board at the Chang Gung Medical Foundation, Taoyuan, Taiwan (Approval No: 201702269B0D001). The procedures were in line with the ethical principles of the Declaration of Helsinki 1975. Before enrollment, all participants provided written informed consent.

**Figure 1:**
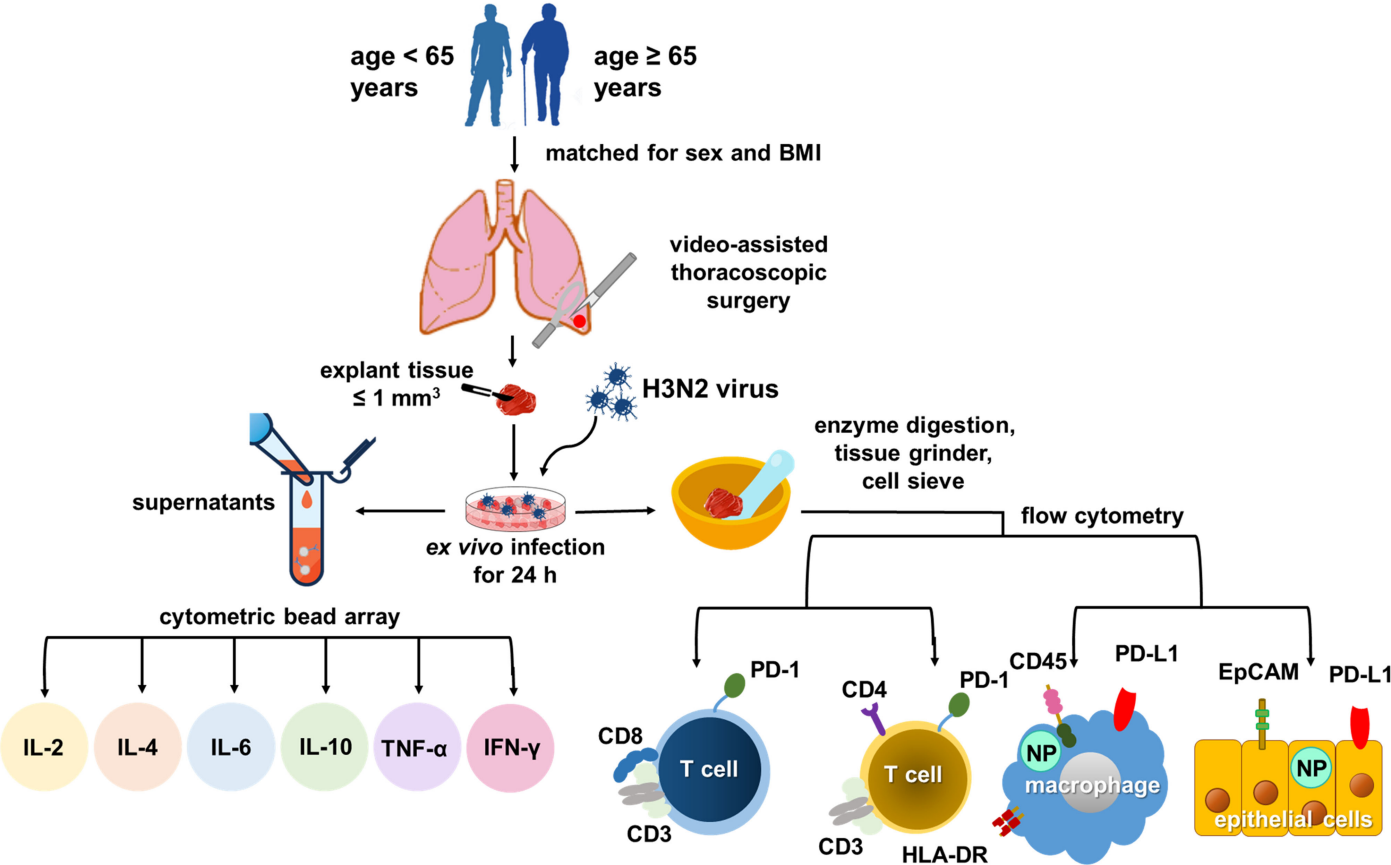
Study flow chart.

We conducted a prospective case-control study. Twelve patients aged 65 years or older (case group) and 24 patients aged less than 65 years (control group) who underwent video-assisted thoracoscopic surgery (VATS) at Chang Gung Memorial Hospital, Linkou Main Branch, Taoyuan, Taiwan, between August 1, 2018, and December 31, 2019, were recruited. Both groups were matched for sex and body mass index (BMI). The study protocol is illustrated in **Figure 1**.

The inclusion criteria for participation were as follows: (1) age above 20 years, (2) undergoing VATS due to suspected lung cancer nodules, and (3) willingness to participate and provide written informed consent. Participants were excluded from the study if they met any of the following criteria: (1) recent respiratory tract viral infection within the last 2 weeks, (2) known history of chronic viral infections such as human immunodeficiency virus, hepatitis B virus, or hepatitis C virus, or (3) known history of interstitial lung disease, severe chronic obstructive lung disease, pulmonary fibrosis, or other lung infectious diseases. Data collected for each participant included age, sex, BMI, history of lung cancer, medical comorbidities (such as hypertension, diabetes, hyperlipidemia, coronary artery disease), and smoking history.

### 2.2 Lung explant tissue

In this study, the primary indication for VATS was the presence of a lung mass. All participants underwent VATS under general anesthesia. After the surgical procedure, human parenchymal tissue with a size of at least 1 cm^3^, which was distant from the resection margin and any gross pathology, was carefully dissected from the lobe. The dissected tissue was then briefly stored in Roswell Park Memorial Institute (RPMI) 1640 medium supplemented with 1% penicillin-streptomycin (both from Life Technologies, Paisley, UK) and 1% gentamicin (GE Healthcare, Little Chalfont, UK) during transport to the laboratory, with a maximum transport time of 30 minutes (Nicholas et al., 2015).

Upon arrival at the laboratory, the tissue was further processed. It was cut into 1-mm^3^ sections, and three pieces were placed into a 24-well flat-bottomed culture plate. Dulbecco’s phosphate-buffered saline (PBS) (Sigma-Aldrich, Poole, UK) was used for washing the tissue three times, followed by a single wash with unsupplemented RPMI 1640 medium. Subsequently, the tissue was incubated overnight at 37°C in a 5% CO_2_ atmosphere using RPMI 1640 medium supplemented with 1% penicillin-streptomycin and 1% gentamicin.

### 2.3 *Ex vivo* infection with H3N2 IAV

The *ex vivo* infection of resected lung tissue with seasonal H3N2 IAV strain A/Texas/50/2012 and mock infection were carried out following established protocols (Nicholas et al., 2015; Staples et al., 2015; Huang et al., 2018). After removing the culture medium, serum-free RPMI was added, supplemented with 100 U/ml penicillin, 100 μg/ml streptomycin, 2 mM/ml L-glutamine, and 250 ng/ml fungizone. Subsequently, the lung explant tissue was exposed to 1×10^6^ plaque-forming units per milliliter of H3N2 IAV or no virus, and incubated at 37°C in a 5% CO_2_ atmosphere for 2 hours. After this initial incubation, the medium was removed, and the tissue was washed with unsupplemented RPMI three times. The lung explant tissue was then incubated for an additional 22 hours in serum-free RPMI supplemented with 100 U/ml penicillin, 100 μg/ml streptomycin, 2 mM/ml L-glutamine, and 250 ng/ml fungizone (Nicholas et al., 2015).

After the designated incubation period, the culture medium underwent centrifugation at 2000 rpm and 4°C for 5 minutes. The resultant supernatants were meticulously collected and promptly stored at -80°C to facilitate subsequent cytokine analysis. Concurrently, the explanted lung tissue was digested using type I collagenase (150 U/mL) and type IIA elastase (10 U/mL), both sourced from Sigma Chemical Co., St. Louis, MO, USA, operating at a temperature of 37°C for a predetermined duration of 15 minutes, as previously detailed in reference (Nicod et al., 1989). Subsequently, the lung tissue was meticulously fragmented using a tissue grinder. The resulting dissociated tissue constituents underwent a gentle transfer through a 35 μm cell sieve, a step that greatly enhanced the efficacy of the isolation procedure. Subsequently, single cells were subjected to a single wash with PBS, employing a centrifugal force of 400 × g for 5 minutes. Subsequently, the lung tissue cells obtained from this digestion were then sent for flow cytometric analysis to assess the immune cell populations and other relevant biomarkers.

### 2.4 Verification of H3N2 IAV infection by viral nucleoprotein

Cells that exhibited immunoreactivity for the IAV nucleoprotein (NP) were determined to be virally infected (Hu et al., 2017; Martinez-Colon et al., 2019). An increased NP expression in target cells was indicative of productive infection (Portela and Digard, 2002). Recognizing that the primary infection site for IAV is the respiratory tract’s epithelial cells, which serve as the principal locale for IAV replication (Dou et al., 2018), along with the understanding that certain IAV strains can productively infect macrophages (Cline et al., 2017), this research focused on the H3N2 IAV infection in both epithelial cells and macrophages. It is also pertinent to mention that pandemic IAV strains have been observed to cause abortive infections in primary human T cells (Yu et al., 2022).

For the identification of NP-positive cells, samples were resuspended in BD Pharmingen stain buffer (catalog No: 554656, Becton Dickinson Biosciences, San Jose, CA, USA) supplemented with heat-inactivated fetal bovine serum (FBS) proteins (incubated at 56°C for 30 minutes). Intracellular staining for viral NP used the BD Cytofix/Cytoperm fixation/permeabilization kit (catalog no. 554714, Becton Dickinson Biosciences, San Jose, CA, USA), following manufacturer instructions by Becton Dickinson Biosciences. Post-fixation and permeabilization, samples were incubated for 30 minutes in stain buffer (FBS) on ice, away from light, to block Fc receptors, minimizing nonspecific antibody binding common in lung macrophages, as detailed in reference (Andersen et al., 2016). During this step, a FITC influenza A NP monoclonal antibody (catalog no. MA1-7322, Thermo Fisher Scientific, Waltham, MA, USA; **Supplementary Table 1**) was added for NP detection. An isotype-matched antibody, sourced from the same manufacturer, served as a specificity control.

To create a baseline, an unstained singlet population was obtained from digested tissue, excluding dead cells using Fixable Viability Stain 700 (Becton Dickinson Biosciences, catalog no. 564997). The resulting lung tissue cell sample was analyzed for intracellular IAV NP expression using the FACSAria IIu Cell Sorter. FACS data were then processed using FACSDiva software v5.0.3. The flow cytometric analysis methodology aligned with standard procedures as outlined in earlier literature.

### 2.5 Detection of epithelial cells, macrophages, CD3^+^/CD4^+^ T Cells, and CD3^+^/CD8^+^ T cells by flow cytometric analysis

During the flow cytometric analysis, the categorization of epithelial cells hinged on the presence of CD45^-^/EpCAM^+^ biomarkers, whereas macrophages were defined by their CD45^+^/HLA-DR^+^ patterns (McKendry et al., 2016). T cells were discerned through their CD45^+^/CD3^+^ phenotype, and within the T cell subset, further distinctions were made between CD3^+^/CD4^+^ T cells and CD3^+^/CD8^+^ T cells. Prior to subjecting the samples to analysis, they were suspended in a stain buffer (FBS). Subsequently, the samples were subjected to a 30-minute incubation on ice in the absence of light, with the introduction of fluorescently labeled antibodies or their respective isotype controls. All antibodies utilized in this process were procured from Becton Dickinson Biosciences, San Jose, CA, USA.

Specific targeting of CD45, EpCAM, HLA-DR, CD3, CD4, and CD8 molecules was accomplished using the following fluorescently labeled antibodies (**Supplementary Table 1**): PerCp-Cy5.5 mouse anti-human CD45 antibody (catalog no. 564105), BV786 mouse anti-human CD326 (EpCAM) antibody (catalog no. 565685), APC-H7 mouse anti-human HLA-DR antibody (catalog no. 561358), FITC mouse anti-human CD3 antibody (catalog no. 561806), APC mouse anti-human CD4 antibody (catalog no. 565994), and PE mouse anti-human CD8 antibody (catalog no. 560959). These antibodies, all from Becton Dickinson Biosciences, were employed in accordance with the stipulations laid out by the manufacturer. Evaluation of the lung tissue cells’ surface expression of these biomarkers was executed utilizing the FACSAria IIu Cell Sorter, as detailed above.

### 2.6 Measurement of TLRs by flow cytometric analysis

The influence of IAV infection on TLR expression has been documented. Specifically, IAV has been observed to increase TLR1–3 and TLR7 mRNA expression in macrophages, while TLR3 mRNA expression is heightened in epithelial cells (Miettinen et al., 2001). Mechanistically, TLR2 is involved in the detection of virus coat proteins and functions in synergy with TLR1 and TLR6. Meanwhile, TLR3 is involved in identifying viral double-strand RNA (Jin and Lee, 2008; Lester and Li, 2014). Expressions of TLRs on the epithelial cells and macrophages were analyzed using flow cytometry. As previously mentioned, samples were resuspended in stain buffer (FBS) and then incubated on ice in the dark for 30 minutes in the presence of fluorescently labeled antibodies or isotype controls. For TLR detection (**Supplementary Table 1**), BV421 mouse anti-human CD281 (TLR1) antibody (catalog no. 566430, Becton Dickinson Biosciences, San Jose, CA, USA) and BV510 mouse anti-human CD282 (TLR2) (catalog no. 742767, Becton Dickinson Biosciences, San Jose, CA, USA), and PE mouse anti-human CD283 (TLR3) antibody (catalog no. 315009, BioLegend, Inc., San Diego, CA, USA) were used. The staining procedure followed the manufacture’s protocols. The surface expressions of TLR molecules on the lung tissue cells were quantified as described in the earlier flow cytometric analysis section.

### 2.7 Measurement of PD-1/PD-L1 pathways by flow cytometric analysis

Flow cytometry was used to assess PD-1 expressions on CD3^+^/CD4^+^ T cells and CD3^+^/CD8^+^ T cells and PD-L1 expressions on epithelial cells and macrophages. After resuspension in stain buffer (FBS), samples were incubated with appropriate fluorescently labeled antibodies or isotype controls, following the manufacturer’s protocols. For PD-1 detection, BV421 mouse anti-human CD279 (PD-1) antibody (catalog no. 562516, Becton Dickinson Biosciences, San Jose, CA, USA) was used, and for PD-L1 detection, APC Mouse anti-human CD274 (PD-L1) antibody (catalog no. 563741, Becton Dickinson Biosciences, San Jose, CA, USA) was used (**Supplementary Table 1**). The expressions of PD-1 and PD-L1 on lung tissue cells were then quantified as described in the earlier section.

### 2.8 Measurement of secreted cytokine levels in culture supernatants

Using a cytometric bead array, we measured levels of interleukin (IL)-2, IL-4, IL-6, IL-10, tumor necrosis factor-alpha (TNF-α), and interferon-gamma (IFN-γ) in the culture supernatants. Each capture bead (Human Soluble Protein Flex Set System, BD Biosciences, San Jose, CA, USA) had distinct fluorescence and was coated with a specific capture antibody. Upon incubation with standards or specimens, sandwich complexes formed and were identified using flow cytometry as outlined in earlier literature (Shetty et al., 2017).

### 2.9 Sample size estimation

Our primary outcome was viral expression at 24 hours post-infection. Sample size was estimated based on a pilot study of *ex vivo* H7N9 IAV infection (Huang et al., 2018). In the pilot study, viral RNA quantities (log transformation) were measured, and the mean values were found to be 5.81 (standard deviation 0.95) for the “aged < 65 years” group and 4.56 (standard deviation 1.37) for the “aged ≥ 65 years” group. The study aimed to achieve 80% power with a significance level (type I error) of 0.05 and an allocation ratio of 0.5. A minimum sample size of 36 participants was deduced, including 24 patients in the “aged < 65 years” group and 12 patients in the “aged ≥ 65 years” group.

### 2.10 Statistical analysis

Given the limited sample size and the nonparametric nature of the variables of interest, which were assessed using the Shapiro-Wilk test, descriptive statistics were presented as median (interquartile range [IQR]) for continuous and skewed variables, and as numbers (proportions) for categorical variables. Percentage (%) difference was calculated using the formula: (value for H3N2 IAV infection group – value for mock infection group)/variable for mock infection group × 100. In this calculation, value of mock infection group served as reference. To ensure that there were no gaps in the assay results and to avoid inaccuracies in estimating concentrations, samples with undetectable values were treated as follows: they were plotted as either 0.1 pg/mL or one-half of the lower limit of quantitation for that particular cytokine, whichever value was greater (Breen et al., 2011).

In the analysis of continuous variables, the Mann-Whitney *U* test was employed to assess differences between two groups (e.g., age ≥ 65 years vs. age < 65 years), while the related-samples Wilcoxon signed-rank test was used to assess within-group differences (e.g., H3N2 IAV infection vs. mock infection) when appropriate. For independent categorical variables, such as age groups, Fisher’s exact test or chi-square test was used to analyze differences between the subgroups when appropriate. To examine relationships between variables of interest, the Spearman correlation test was utilized. In comparing differences in outcome variables by age group (age ≥ 65 years vs. age < 65 years), generalized estimating equations were applied with adjustments for potential confounding factors (such as sex, obesity, lung cancer history, medical comorbidity history, and smoking history).

In the *post hoc* analysis, the researchers evaluated the profiles of TLRs, PD-1/PD-L1 pathways, viral expressions, and cytokines. To identify the regulation patterns of these variables, unsupervised cluster analysis was conducted based on the % differences of the 18 variables of interest. The hierarchical cluster analysis algorithm was used to determine the number of clusters and assess their robustness. Subsequently, between-group comparisons were performed using the independent-samples Kruskal-Wallis test to examine if there were significant differences among the identified clusters. Given the multiple tests conducted, to control for the increased risk of type I error, the significant *P*-values obtained were adjusted using the Bonferroni correction method.

All statistical calculations are performed with G*Power 3.1.9.2 (Heinrich-Heine University, Dusseldorf, Germany), SPSS (version 27; International Business Machines Corp., Armonk, NY, USA), and GraphPad Prism for Windows (version 10.0; GraphPad Software, Inc., San Diego, CA) software packages. A two-sided *P*-value of < 0.05 was considered statistically significant.

## 3 Results

### 3.1 Patient characteristics

**Figure 2:**
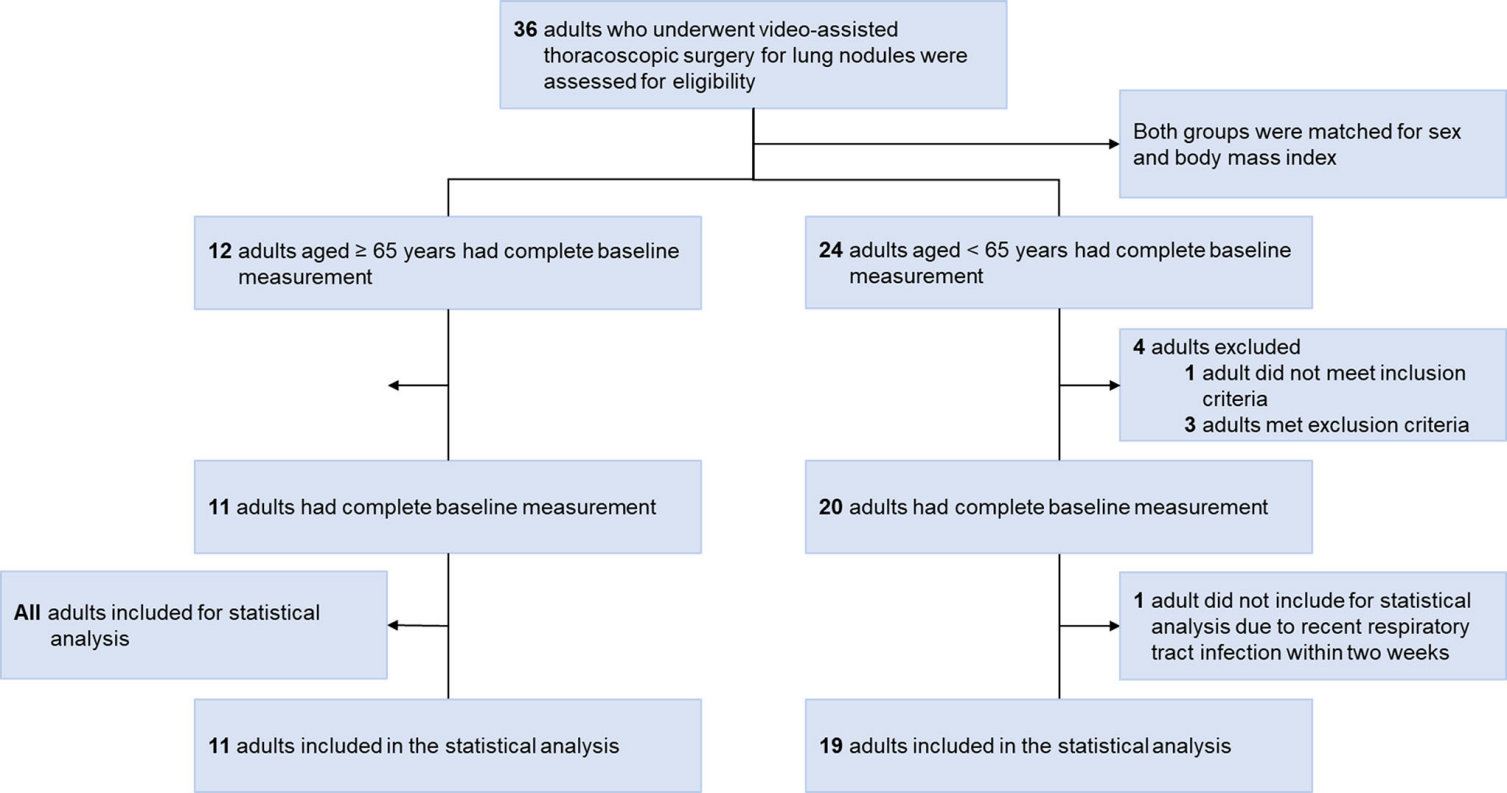
Flow diagram. Thirty-six Taiwanese adults with lung nodules who met the inclusion and exclusion criteria underwent video-assisted thoracoscopic surgery during the study period. Five patients were initially excluded; one who was unwilling to participate before video-assisted thoracoscopic surgery, and four who had contraindications. Additionally, one was further excluded due to a recent respiratory tract infection. Therefore, 14 (47%) men and 16 (53%) women were included in the statistical analysis.

**Figure 2** provides a flowchart detailing the recruitment and analysis of participants. Out of the 36 Taiwanese adults with lung nodules who underwent VATS, five were excluded from analysis. Additionally, one was excluded due to a recent respiratory tract infection. Therefore, the analysis included 30 participants: 14 males and 16 females. Their median age was 63 years, with a median BMI of 23.9 kg/m^2^. **Table 1** provides a detailed breakdown of their demographic data. It is noteworthy that patients aged ≥ 65 years had a higher prevalence of medical comorbidities compared to those aged < 65 years.

**Table 1:** Clinical characteristics of the study participants.

Variables	Total	Age ≥ 65 years	Age < 65 years	*P*-Value
Number of patients	30	11	19	
Age, year	63 (56–67)	**69 (66–75)**	**58 (53–61)**	< 0.001^b^
Men/women	14/16	5/6	9/10	> 0.999^c^
Body mass index, kg/m^2^	23.9 (22.2–25.7)	24.5 (22.6–30.0)	23.6 (21.9–24.6)	0.368^b^
Obesity, no/yes	24/6	7/4	17/2	0.156^b^
Lung cancer history, no/yes	14/16	4/7	10/9	0.466^c^
Medical comorbidity history^a^, no/yes	17/13	**3/8**	**14/5**	0.023^c^
Smoker, never/*ex*/current	23/1/6	9/0/2	14/1/4	0.716^c^

Results are presented as median (interquartile range) for continuous variables and as absolute number for categorical variables.

Bold font indicates statistically significant differences (P < 0.05).

a Medical comorbidities included hypertension (n = 6), diabetes mellitus (n = 2), hyperlipidemia (n = 4), and coronary artery disease (n = 2).

b Continuous variables of the “age ≥ 65 years” group were compared with the “age < 65 years” group using the Mann-Whitney U test.

c Categorical variables of the “age ≥ 65 years” subgroup were compared with the “age < 65 years” subgroup using Fisher’s exact test or the chi-square test, as appropriate.

### 3.2 Molecular expression in lung explant tissue

During the early phase of H3N2 IAV infections, a distinct upsurge in NP expression was observed in both epithelial cells and macrophages of the H3N2-infected cohort, relative to the mock-infected group (**Table 2**). Particularly, 24 hours following infection, the macrophages from the H3N2-infected group manifested a pronounced rise in PD-L1 expression in contrast to those from the mock-infected group.

**Table 2:** Difference in the expressions of molecular biomarkers in immune cells and secreted cytokines in culture supernatants between mock infection and influenza A(H3N2) infection groups after 24 h post-infection.

Molecular Biomarkers	H3N2 infection	Mock infection	% Differences	*P*-Value^a^
Number of patients	30	30		
CD45^-^/EpCAM^+^ epithelial cells				
NP, %	**1.91 (1.12–6.10)**	**1.25 (0.09–8.00)**	**52 (2–700)**	**0.022**
TLR1, %	3.83 (1.14–9.43)	4.35 (1.56–11.92)	23 (-31–100)	0.636
TLR2, %	2.07 (0.64–5.52)	2.01 (0.28–5.82)	21 (-17–92)	0.144
TLR3, %	2.15 (1.00–3.95)	1.62 (0.90–3.56)	14 (-14–63)	0.148
PD-L1, %	3.60 (0.83–9.87)	2.57 (0.04–7.53)	22 (-20–111)	0.059
CD45^+^/HLA-DR^+^ macrophages				
NP, %	**2.42 (0.81–11.60)**	**1.30 (0.31–8.28)**	**109 (21–212)**	**0.003**
TLR1, %	2.20 (0.47–7.40)	1.58 (0.45–6.77)	12 (-31–152)	0.370
TLR2, %	2.34 (0.78–12.81)	2.59 (0.99–15.46)	-18 (-41–54)	0.750
TLR3, %	0.81 (0.38–3.98)	0.71 (0.29–3.28)	36 (-41–103)	0.236
PD-L1, %	**1.41 (0.49–4.35)**	**0.33 (0.05–1.65)**	**149 (23–681)**	**< 0.001**
CD3^+^/CD4^+^ T cells				
PD-1, %	23.08 (9.67–34.18)	21.75 (8.14–31.77)	6 (-16–16)	0.229
CD3^+^/CD8^+^ T cells				
PD-1, %	21.01 (12.60–30.65)	19.91 (12.20–27.75)	2 (-7–18)	0.109
Secreted cytokines in culture supernatants				
IL-2, pg/mL	**0.10 (0.10–8.66)**	**0.10 (0.10–0.10)**	**0 (0–3230)**	**0.001**
IL-4, pg/mL	0.10 (0.10–0.10)	0.10 (0.10–0.10)	0 (0–0)	0.285
IL-6, pg/mL	**8210 (3651–21704)**	**3481 (1818–13182)**	**44 (-3–247)**	**0.008**
IL-10, pg/mL	**8.86 (1.77–19.32)**	**1.09 (0.10–8.54)**	**241 (0–4518)**	**0.004**
TNF-α, pg/mL	**45.61 (27.36–88.02)**	**3.24 (0.10–13.02)**	**1045 (292–7620)**	**< 0.001**
IFN-γ, pg/mL	**20.16 (7.87–49.23)**	**0.10 (0.10–0.10)**	**16375 (2540–33450)**	**< 0.001**

Results are presented as median (interquartile range) for continuous variables.

Bold font indicates statistically significant differences (P < 0.05).

a Within-group comparisons were performed using the related-samples Wilcoxon signed-rank test for continuous variables.

CD, cluster of differentiation; EpCAM, epithelial cell adhesion molecule; HLA-DR, human leukocyte antigen-DR; IFN-γ, interferon-gamma; IL, interleukin; NP, nucleoprotein; PD-1, programmed death 1; PD-L1, programmed death-ligand 1; TLR, toll-like receptor; TNF-α, tumor necrosis factor-alpha.

**Figure 3:**
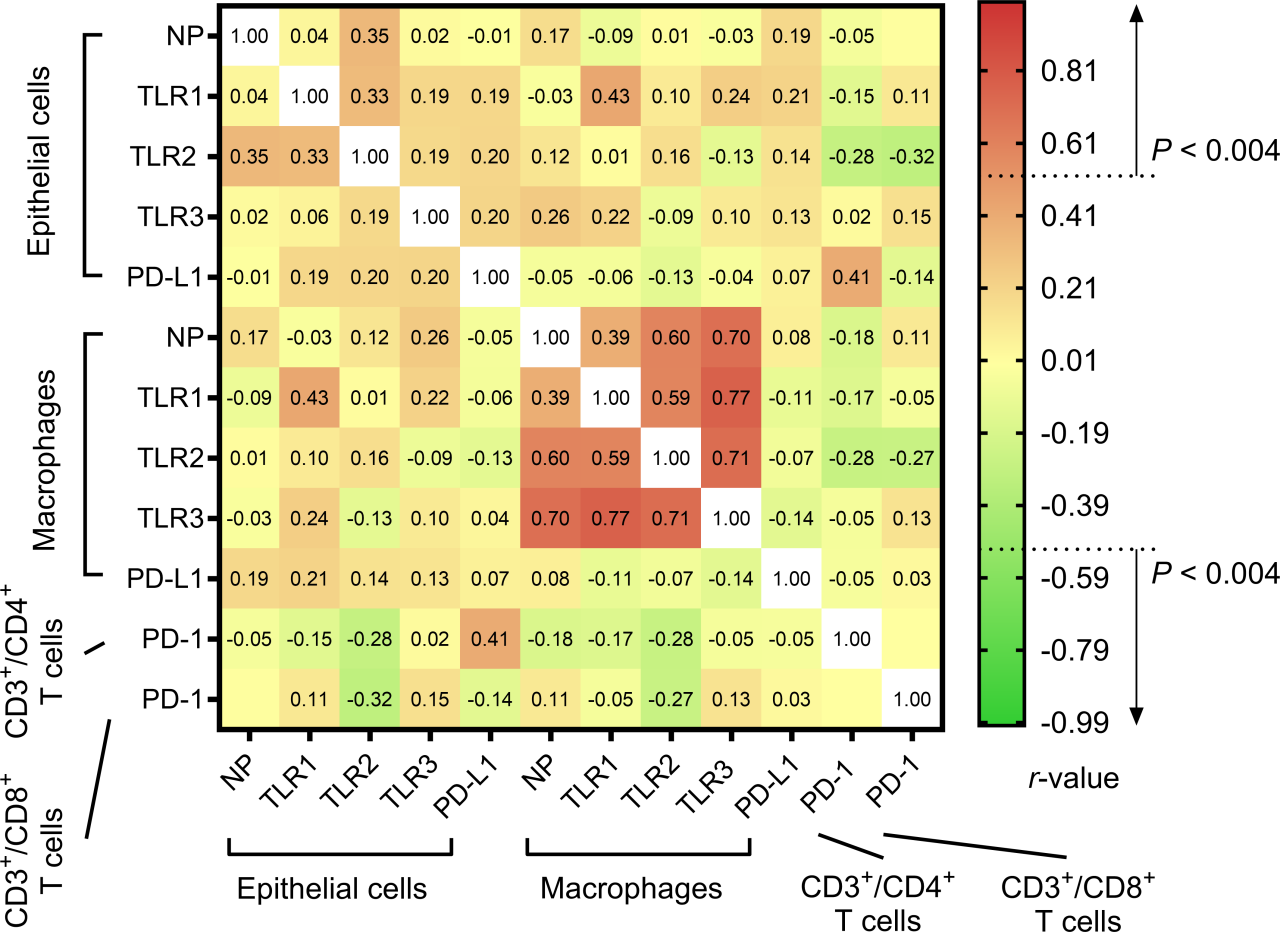
Heatmap of the relationships between % differences in the expressions of molecular markers in lung tissue cells. Their associations were assessed using the Spearman correlation test and presented as *rho*.

**Figure 3** elucidates the intricate interplay between different molecular markers. Initial assessments spotlighted a robust positive relationship between the % difference in TLR1 expression across epithelial cells and macrophages. Similarly, a strong association was detected between % differences in PD-L1 expression on epithelial cells and PD-1 expression on CD3^+^/CD4^+^ T cells. Nevertheless, these initial relationships lost their statistical significance post the application of the Bonferroni correction. In contrast, correlations concerning % differences in NP and TLR1–3 expressions in macrophages retained their significance post-adjustment.

### 3.3 Secreted cytokine levels in culture supernatants

There was a significant increase in in the secretion of cytokines IL-2, IL-6, IL-10, TNF-α, and IFN-γ in the H3N2 virus-infected group 24 hours after infection (**Table 2**). **Table 3** highlights correlations between cytokine secretion and molecular marker expression. Importantly, the % difference in secreted IL-2 level was directly correlated with % differences in TLR1 expression on epithelial cells and PD-L1 expression on macrophages. Additionally, significant inverse relationships were observed between the % difference in secreted IL-6 level and % difference in TLR2 expression on macrophages, as well as between the % difference in secreted IL-10 level and % difference in TLR1 expression on macrophages. However, these relationships lost their statistical significance after the Bonferroni correction.

**Table 3:** Associations of % differences in nucleoprotein (NP), toll-like receptors (TLRs) and programmed death 1 (PD-1)/programmed death-ligand 1 (PD-L1) pathways with % differences in secreted cytokine levels.

% Differences	IL-2	IL-4	IL-6	IL-10	TNF-α	IFN-γ
CD45^-^/EpCAM^+^ epithelial cells						
NP	-0.02 (0.899)	0.297 (0.111)	0.11 (0.576)	0.04 (0.833)	0.32 (0.083)	0.16 (0.394)
TLR1	**0.39 (0.031**)	-0.04 (0.817)	0.02 (0.910)	-0.21 (0.257)	0.23 (0.215)	0.17 (0.384)
TLR2	-0.09 (0.630)	0.06 (0.755)	0.19 (0.318)	-0.18 (0.344)	0.18 (0.342)	-0.04 (0.826)
TLR3	-0.01 (0.980)	0.33 (0.078)	-0.07 (0.721)	0.04 (0.841)	-0.28 (0.130)	-0.10 (0.596)
PD-L1	-0.08 (0.692)	-0.03 (0.891)	0.25 (0.175)	0.16 (0.390)	0.06 (0.753)	0.26 (0.163)
CD45^+^/HLA-DR^+^ macrophages						
NP	-0.21 (0.257)	0.29 (0.117)	-0.20 (0.302)	-0.23 (0.219)	-0.11 (0.569)	-0.30 (0.105)
TLR1	-0.22 (0.255)	0.23 (0.219)	-0.31 (0.095)	**-0.37 (0.047)**	0.02 (0.914)	-0.11 (0.555)
TLR2	-0.21 (0.276)	0.05 (0.810)	**-0.41 (0.025)**	-0.20 (0.293)	-0.02 (0.925)	-0.17 (0.367)
TLR3	-0.18 (0.353)	0.20 (0.280)	-0.33 (0.079)	-0.25 (0.178)	0.01 (0.986)	-0.13 (0.483)
PD-L1	**0.41 (0.026)**	-0.16 (0.407)	0.16 (0.412)	-0.22 (0.251)	-0.15 (0.438)	-0.23 (0.223)
CD3^+^/CD4^+^ T cells						
PD-1	-0.03 (0.864)	-0.27 (0.151)	0.01 (0.980)	0.09 (0.643)	-0.04 (0.844)	0.14 (0.448)
CD3^+^/CD8^+^ T cells						
PD-1	0.19 (0.322)	0.07 (0.718)	0.04 (0.847)	0.14 (0.452)	0.09 (0.629)	0.01 (0.979)

Results are presented as *rho* (P-value).

Bold font indicates statistically significant differences (P < 0.05).

Relationships between variables of interest were assessed using the Spearman correlation test.

CD, cluster of differentiation; EpCAM, epithelial cell adhesion molecule; HLA-DR, human leukocyte antigen-DR; IFN-γ, interferon-gamma; IL, interleukin; TNF-α, tumor necrosis factor-alpha.

### 3.4 Age-related variations adjusted for confounding factors

Initial assessment indicated only a handful of variables exhibiting age-dependent variations (**Table 4**). Notably, the expression of TLR2 on macrophages showcased age-based differences, which were neutralized upon adjusting for confounders. In a post-adjustment scenario, individuals aged ≥ 65 years showed decreased secretions of IL-2 and IFN-γ after H3N2 virus infection, compared to those aged < 65 years. Age did not exhibit a pronounced effect on TLR signaling or the PD-1/PD-L1 pathways.

**Table 4:** Comparison of % differences in the variables of interest between the age ≥ 65 years group and age < 65 years group.

Molecular Biomarkers	Age ≥ 65 years	Age < 65 years	Unadjusted *P*-value^a^	Adjusted *P*-value^b^	β (95% CI)
	Factor	Reference			
Number of patients	11	19			
CD45^-^/EpCAM^+^ epithelial cells					
NP	7 (-29–160)	242 (12–700)	0.158	0.850	
TLR1	-15 (-59–100)	24 (-28–100)	0.525	0.223	
TLR2	-1 (-51–100)	32 (5–89)	0.307	0.952	
TLR3	-6 (-14–49)	34 (-11–70)	0.287	0.272	
PD-L1	60 (-18–180)	16 (-31–47)	0.268	0.286	
CD45^+^/HLA-DR^+^ macrophages					
NP	123 (67–289)	85 (-4–186)	0.553	0.419	
TLR1	13 (-12–168)	8 (-47–150)	0.641	0.070	
TLR2	**50 (-20–178)**	**-23 (-46–17)**	**0.021**	0.460	
TLR3	80 (0–153)	0 (-47–95)	0.200	0.267	
PD-L1	220 (0–600)	104 (51–925)	0.832	0.797	
CD3^+^/CD4^+^ T cells					
PD-1	18 (-7–111)	3 (-19–13)	0.064	0.283	
CD3^+^/CD8^+^ T cells					
PD-1	-7 (-19–11)	2 (-2–22)	0.216	0.121	
Secreted cytokines in the culture supernatants					
IL-2	**0 (0–4880)**	**15 (0–2680)**	0.420	**0.002**	**-15 (-24–5)**
IL-4	0 (0–0)	0 (0–0)	0.420	0.881	
IL-6	13 (-16–125)	61 (0–509)	0.145	0.501	
IL-10	161 (0–1890)	391 (0–5100)	0.611	0.896	
TNF-α	535 (172–4700)	1455 (474–28310)	0.094	0.364	
IFN-γ	**17580 (3154–28980)**	**14750 (700–33690)**	0.800	**0.003**	**-38 (-63–13)**

Results are presented as median (interquartile range) for continuous variables.

Bold font indicates statistically significant differences (P < 0.05).

a % Differences in the variables of interest in the “age ≥ 65 years” group were compared with those in the “age < 65 years” group using the Mann-Whitney U test.

b Data were further compared using generalized estimating equation analysis adjusted for sex, obesity, lung cancer history, medical comorbidity history, and smoking history.

CD, cluster of differentiation; EpCAM, epithelial cell adhesion molecule; HLA-DR, human leukocyte antigen-DR; IFN-γ, interferon-gamma; IL, interleukin; NP, nucleoprotein; PD-1, programmed death 1; PD-L1, programmed death-ligand 1; TLR, toll-like receptor; TNF-α, tumor necrosis factor-alpha.

### 3.5 *Post Hoc* analysis

**Figure 4:**
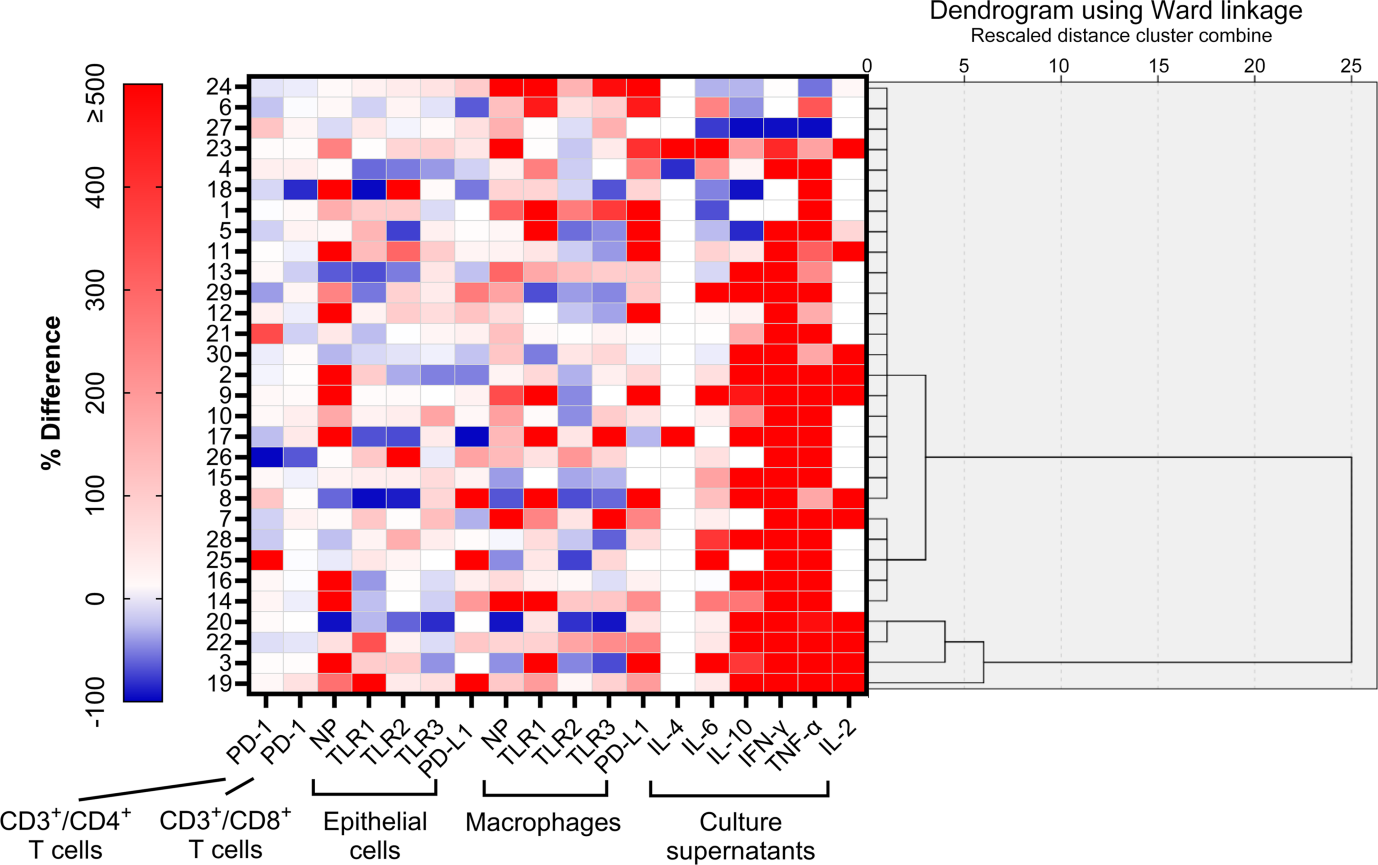
Unsupervised clustering analysis of regulation patterns according to % differences in 18 variables of interest. Using a hierarchical cluster analysis algorithm, three clusters were identified.

**Figure 5:**
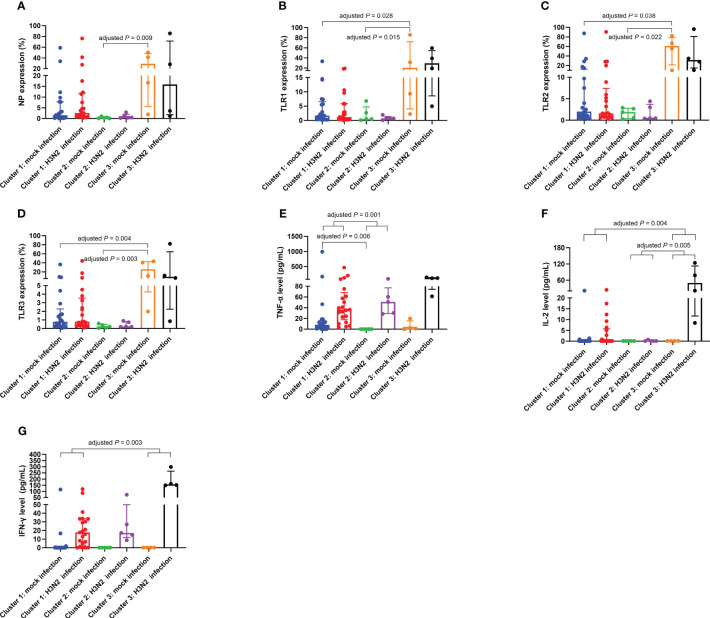
Differential expressions of NP **(A)**, TLR1 **(B)**, TLR2 **(C)**, and TLR3 **(D)** on macrophages, TNF-α levels in culture supernatants **(E)**, IL-2 levels in culture supernatants **(F)**, and IFN-γ levels in culture supernatants **(G)** across Cluster 1, Cluster 2, and Cluster 3. The presented data are represented as medians with interquartile ranges. Between-group comparisons were conducted using the independent-samples Kruskal-Wallis test with Bonferroni correction. IFN-γ, interferon-gamma; IL, interleukin; NP, nucleoprotein; TLR, toll-like receptor; TNF-α, tumor necrosis factor-alpha.

Unsupervised clustering led to the classification of participants into three discernible clusters: Cluster 1 (*n* = 21), Cluster 2 (*n* = 5), and Cluster 3 (*n* = 4), detailed in **Figure 4**. Using molecular marker expressivity and post-mock infection cytokine dynamics as baselines, this analysis revealed that patients in Cluster 3 had maximal expressions of NP and TLR1–3 within macrophages (**Figures 5A–D**). In contrast, those in Cluster 1 had a significant increase in TNF-α secretions (**Figure 5E**) compared to Cluster 2 participants (**Table 5**).

**Table 5:** Comparisons of clinical characteristics and variables of interest after mock infection across the three clusters.

Variables	Cluster 1	Cluster 2	Cluster 3	*P*-value^a^
Number of patients	21	5	4	
Age, year	64 (58–69)	58 (37–69)	60 (55–65)	0.394
Men/women	9/12	2/3	3/1	0.472
Body mass index, kg/m^2^	23.9 (22.1–25.0)	24.4 (20.4–28.5)	23.4 (22.1–26.6)	0.982
Obesity, no/yes	4/17	1/4	1/3	0.963
Lung cancer history, no/yes	14/7	1/4	1/3	0.081
Medical comorbidity history, no/yes	10/11	1/4	2/2	0.512
Smoker, never/*ex*/current	17/1/3	4/0/1	2/0/2	0.558
CD45^-^/EpCAM^+^ epithelial cells				
NP expression, %	1.30 (0.20–9.32)	1.91 (0.05–16.45)	4.2 (0.06–1.27)	0.503
TLR1 expression, %	3.11 (1.52–10.09)	9.70 (4.39–21.14)	6.67 (0.39–12.04)	0.332
TLR2 expression, %	0.80 (0.20–4.80)	4.34 (1.80–13.10)	7.60 (0.95–40.93)	0.242
TLR3 expression, %	1.40 (0.78–2.58)	2.03 (0.67–6.12)	3.41 (1.53–31.67)	0.314
PD-L1 expression, %	1.70 (0.30–3.95)	7.24 (3.16–33.31)	6.73 (0.46–52.21)	0.213
CD45^+^/HLA-DR^+^ macrophages				
NP expression, %	1.55 (0.34–7.76)	0.27 (0.07–0.75)^c^	28.97 (5.61–47.97)^c^	**0.012**
TLR1 expression, %	1.20 (0.37–5.82)^b^	0.70 (0.40–1.39)^c^	**29.27 (8.58–54.40)^b,c^**	**0.012**
TLR2 expression, %	2.00 (0.98–12.26)^b^	1.90 (0.38–2.79)^c^	**61.08 (21.50–78.29)^b,c^**	**0.019**
TLR3 expression, %	0.80 (0.32–2.29)^b^	0.23 (0.07–0.50)^c^	**25.86 (4.27–42.60)^b,c^**	**0.004**
PD-L1 expression, %	0.10 (0.05–0.70)	0.93 (0.05–2.94)	2.17 (0.48–36.33)	0.172
CD3^+^/CD4^+^ T cells				
PD-1 expression, %	22.60 (8.56–32.94)	19.93 (4.19–26.48)	11.08 (6.38–64.70)	0.517
CD3^+^/CD8^+^ T cells				
PD-1 expression, %	20.50 (10.99–28.00)	20.02 (16.03–27.45)	13.03 (11.69–42.18)	0.821
Secreted cytokines in the culture supernatants				
IL-2, pg/mL	0.10 (0.10–0.10)	0.10 (0.10–0.10)	0.10 (0.10–0.10)	0.367
IL-4, pg/mL	0.10 (0.10–0.10)	0.10 (0.10–0.10)	0.10 (0.10–0.10)	0.807
IL-6, pg/mL	3423 (2008–12107)	4705 (740–14006)	8101 (2282–15747)	0.898
IL-10, pg/mL	1.40 (0.10–10.01)	0.10 (0.10–9.55)	2.94 (0.65–4.54)	0.457
TNF-α, pg/mL	8.14 (2.56–15.87)^b^	0.10 (0.10–0.10)^b^	1.61 (0.30–15.42)	**0.008**
IFN-γ, pg/mL	0.10 (0.10–0.10)	0.10 (0.10–0.10)	0.10 (0.10–0.10)	0.387

Results are presented as median (interquartile range) for continuous variables and as absolute number for categorical variables.

Bold font indicates statistically significant differences (P < 0.05).

a Continuous variables among the three clusters were subjected to comparison using the Kruskal-Wallis test, while categorical variables were compared using the chi-square test across the same clusters.

b Statistical significance at P < 0.05 when comparing Cluster 1 to Cluster 2 or Cluster 1 to Cluster 3.

c Statistical significance at P < 0.05 when comparing Cluster 2 to Cluster 3.

CD, cluster of differentiation; EpCAM, epithelial cell adhesion molecule; HLA-DR, human leukocyte antigen-DR; IFN-γ, interferon-gamma; IL, interleukin; TNF-α, tumor necrosis factor-alpha.

A deeper examination showed distinct variations across clusters in terms of % changes of IL-2, TNF-α, and IFN-γ secretion levels in culture supernatants (**Table 6**). Notably, Cluster 3 had an elevated % difference in secreted IL-2 level (**Figure 5F**) in comparison to Cluster 2. Conversely, Cluster 1 displayed decreased % differences in both secreted IL-2 (**Figure 5F**) and IFN-γ (**Figure 5G**) levels compared to Cluster 3, with a lesser % difference in secreted TNF-α level relative to Cluster 2 (**Figure 5E**).

**Table 6:** Comparative analysis of the % differences in the variables of interest among the three clusters.

Molecular Biomarkers	Cluster 1	Cluster 2	Cluster 3	*P*-value^a^
Number of patients	21	5	4	
CD45^-^/EpCAM^+^ epithelial cells				
NP	43 (6–667)^d^	9 (-17–1560)	170 (-56–595)	0.979
TLR1	7 (-56–71)	22 (-33–78)	220 (4–594)	0.215
TLR2	21 (-42–95)	5 (2–89)	34 (-39–85)	0.945
TLR3	28 (-7–68)^d^	0 (-16–81)	-28 (-73–43)	0.196
PD-L1	20 (-24–81)	32 (-12–3241)	55 (0–483)	0.523
CD45^+^/HLA-DR^+^ macrophages				
NP	124 (27–243)^d^	70 (-25–2994)	21 (-81–102)	0.210
TLR1	13 (-26–155)	11 (-32–266)	21 (-78–112)	0.675
TLR2	-20 (-37–56)	13 (-49–80)	-15 (-73–138)	0.882
TLR3	31 (-38–100)	78 (-38–906)	9 (-87–191)	0.675
PD-L1	104 (6–1200)^d^	68 (13–230)	219 (87–755)	0.562
CD3^+^/CD4^+^ T cells				
PD-1 expression	3 (-17–20)	15 (-20–956)	7 (-9–13)	0.821
CD3^+^/CD8^+^ T cells				
PD-1 expression	5 (-7–18)	-1 (-4–13)	3 (-7–45)	0.924
Secreted cytokines in the culture supernatants				
IL-2	**0 (0–1652)^b,d^**	0 (0–305)^c^	**49370 (11508–112373)^b,c^**	0.003
IL-4	0 (0–0)	0 (0–0)	0 (0–0)	0.926
IL-6	31 (-21–198)	263 (16–451)	44 (34–809)	0.358
IL-10	161 (-15–3425)	267 (0–10745)	886 (421–50708)	0.271
TNF-α	**609 (177–1357)^b,d^**	**50620 (29235–77005)^b,d^**	5937 (1609–108733)	< 0.001
IFN-γ	**8070 (212–29535)^b,d^**	17050 (11710–49755)^d^	156615 (149985–263873)^b^	0.004

Results are presented as median (interquartile range) for continuous variables.

Bold font indicates statistically significant differences (P < 0.05).

a % Differences in the variables of interest across the three clusters were compared using the independent-samples Kruskal-Wallis test with Bonferroni correction.

b Statistical significance at P < 0.05 when comparing Cluster 1 to Cluster 2 or Cluster 1 to Cluster 3.

c Statistical significance at P < 0.05 when comparing Cluster 2 to Cluster 3.

d Statistical significance at P < 0.05 when related-samples Wilcoxon signed-rank test was used to assess differences between the H3N2 IAV infection and the mock infection.

CD, cluster of differentiation; EpCAM, epithelial cell adhesion molecule; HLA-DR, human leukocyte antigen-DR; IFN-γ, interferon-gamma; IL, interleukin; NP, nucleoprotein; PD-1, programmed death 1; PD-L1, programmed death-ligand 1; TLR, toll-like receptor; TNF-α, tumor necrosis factor-alpha.

## 4 Discussion

The findings from our investigation amplify the immense value of utilizing lung explant tissue cultures as an advanced model to dissect the complexities inherent to viral infections. Prioritizing human explant tissues in our approach has enriched our perspective, casting a wider net over immunological dynamics that encompasses a myriad of immune cells and their respective microenvironments. This level of granularity and breadth has been notably absent in earlier studies.

In the context of *in vivo* infections, the IAV, as corroborated by previous research, predominantly targets airway epithelial cells initially (Portela and Digard, 2002). This targeting is directly responsible for a cascade of events leading to viral replication, its subsequent release, and the eventual manifestation of clinical symptoms. The next crucial line of defense, the airway macrophages, which are at the heart of innate immunity, are quick to identify and counter the advancing IAV infection (Meischel et al., 2020). What makes our study stand out is how we exposed both these airway cells and the macrophages to the H3N2 virus at the same time. This unique approach helps us understand better how the body’s initial defense mechanisms, especially the activation of certain macrophages, respond to the virus. Our method offers a promising way to study how the virus affects humans, how our bodies react to it, and how to develop treatments against the H3N2 virus, potentially reducing the severity of flu symptoms (Hocke et al., 2017). This model can also help improve the way we test new treatments for their effectiveness against the virus. Moving forward, we’ll explore more about how the immune system responds, how the virus behaves in different immune cells, and how individual differences in patients can change these results.

T cells hold a pivotal position in the intricate tapestry of our body’s immune defense mechanisms. Triggered into action by antigenic presence, they not only mobilize to the lungs but also synthesize key cytokines, predominantly IL-2 and IFN-γ. These cytokines have a propensity to amplify cellular proliferation and augment other cellular functionalities, which can cascade into inflammatory responses (Jagtap, 2018). Our investigative journey illuminated the intricate interplay between TLRs, the PD-1/PD-L1 pathways, and their influence on cytokine concentrations and viral NP expressions within immune cells. A salient revelation from our exploration was the amplified PD-L1 expression in macrophages during the nascent stages of H3N2 IAV infection, which harmonizes with findings from a preceding study (Staples et al., 2015). Of noteworthy mention was the discerned positive association between the variations in PD-L1 expression on epithelial cells and the corresponding variations in PD-1 expression on CD3^+^/CD4^+^ T cells. The magnitude of this association’s importance cannot be overstated. Given that lung epithelial cells double up as both anatomical barricades and antigen presenters, their PD-L1 expression interacts with PD-1, imposing a regulatory mechanism on lung CD4^+^ resident memory T cells, thus modulating barrier immunity (Shenoy et al., 2021). Viral onslaughts, more often than not, excessively stimulate the PD-1/PD-L1 pathways, which could attenuate T cell efficiency (Patil et al., 2017). A scenario wherein these T cells become overburdened, or “exhausted,” could compromise their combative efficacy against viral entities (Wherry, 2011). Hence, maintaining an equilibrium in the PD-1/PD-L1 pathway dynamics among epithelial cells, macrophages, and T cells, especially during the incipient phases of a H3N2 IAV infection, is of paramount significance. This equilibration ensures that our immune response remains vigilant and potent. The observed diminution in the secretions of IL-2 and IFN-γ among the geriatric demographic posits that PD-L1-expressing macrophages might wield greater influence in the phenomena of inflamm-aging (Sharma, 2021).

Our study highlights the relationship between immune responses and the interaction of IL-2 with the PD-1/PD-L1 pathways during H3N2 IAV infections. In simple terms, IL-2, distinguished by its dual pro-inflammatory and anti-inflammatory characteristics, stands out as an influential protagonist in the resistance against microbial incursions (Alam et al., 2020). In the setting of our study with human lung specimens, IL-2 unveiled its multifaceted role in orchestrating immune responses. It promotes the proliferation and potency of CD8^+^ cytotoxic T cells and natural killer cells, which are indispensable components of our immunological arsenal (Kamimura and Bevan, 2007). Concurrently, IL-2 has implications on regulatory T cells, ensuring a check on the immune system, and averting undue inflammatory reactions (Malek and Bayer, 2004). However, a compelling observation emanating from our study was the incongruity between IL-2 secretory levels and viral NP expression. This hints at the possibility that the orchestration of IL-2 secretion may be governed by external determinants, potentially tethered to T cell operational dynamics, especially in scenarios where macrophages manifest heightened PD-L1 expression. Rampant stimulation of the PD-1/PD-L1 pathway is intrinsically linked with T cell malfunction, culminating in compromised antiviral responses across a spectrum of viral incursions (Schonrich and Raftery, 2019; Zhou et al., 2019). Thus, the delicate balance between IL-2 concentrations and PD-L1 levels potentially plays a decisive role in steering T cell behavior amidst H3N2 IAV onslaughts.

A crucial context to consider here is that a considerable fraction of our tissue samples originated from patients diagnosed with lung cancer. The inherent characteristics of the tumor milieu must be factored into our interpretations. It is conceivable that within such a milieu, IL-2 is a harbinger signaling T cell exhaustion (Liu et al., 2021). However, there’s a glimmer of hope, as interventions inhibiting PD-L1 have showcased potential in revitalizing these fatigued T cells, potentially mitigating viral ramifications in certain infection contexts (West et al., 2013). We are thus led to postulate that a synthesis of elevated IL-2 levels combined with suppressed PD-L1 might be conducive for efficient viral clearance during the advanced phases of H3N2 IAV infections. To encapsulate, our findings point to the importance of IL-2 in the body’s fight against viral infections like H3N2 IAV. This molecule, with its dual roles, could be a promising target for future treatments, especially for influenza.

When juxtaposing our results derived from human lung tissue *ex vivo* infection models with those from animal studies, distinct differences emerge. For instance, in rat models, exposure to IAV antigens leads to an escalation in PD-1 expression in innate immune cells during acute lower respiratory tract infections. Concurrently, there’s a rise in PD-L1 expression, which appears to inhibit the action of CD8^+^ T cells (Erickson et al., 2012). Also, elevated levels of IFN-γ are linked to decelerated H3N2 IAV replication in these models, which might explain the subdued immune reactions commonly observed in the elderly (Hernandez-Vargas et al., 2014). However, our *ex vivo* model with human lung tissue provides potentially clarifying insights into these disparities. The prevalent presence of viral NP memory T cells – distinguished by their abundant multifunctional T cells and amplified cytokine output in adult human lungs – might be shaping the immune reactions we detected (Pen et al., 2014). Indications from our model hint at an accelerated release of inflammation-triggering factors steered by PD-L1 after H3N2 IAV infection compared to what has been observed in alternative models (Zhang J. et al., 2020). While animal studies have undeniably added immense value to our knowledge pool, they inherently have limitations in replicating the multifaceted nature of human immune responses. This is where our *ex vivo* model stands out. It offers an authentic window into human-specific reactions, enabling a direct examination of how human lung tissue counteracts H3N2 IAV infection. In doing so, our study illuminates the nuanced choreography of the immune system during human infections, potentially providing a foundation for more tailored therapeutic interventions.

In this study, several illuminating observations came to the fore. One of the most striking was the significant variability we observed in the % difference of PD-L1 expression on macrophages. Another intriguing finding was the correlation of aging with a muted increase in cytokines such as IL-2 and IFN-γ after controlling for potential confounding variables. However, aging was not found to be linked with TLR signaling or the PD-1/PD-L1 pathway. While earlier research posited that the decreased ability of CD8^+^ T cells to combat influenza in the elderly wasn’t due to a decrease in the frequency of virus-specific T cells (Boon et al., 2002), our findings hint at IL-2 secretion playing a possible role. This could be a contributing factor to the observed age-related diminishment in the effectiveness of CD8^+^ T cells against the H3N2 IAV, as reported in preceding studies (Zhou and McElhaney, 2011; Zhou et al., 2016). Consequently, our research adds a crucial layer of understanding to the evolving dialogue on age-related immunosenescence (Yoshikawa, 2000; Rodrigues et al., 2021). It underscores the importance of recognizing the delicate and complex balance of immune responses, especially against pathogens like the H3N2 IAV, and how these responses evolve with age. The revelations from this study not only inform future research directions but also point toward potential interventions to bolster immune responses, especially in our aging population.

Our detailed exploration into the innate immune responses and inflammasome activation post-*ex vivo* IAV infection has revealed intriguing patterns. By delineating our data into three distinct clusters: Cluster 1 (intermediate NP expression in macrophages and high secreted TNF-α level), Cluster 2 (low NP expression in macrophages and low secreted TNF-α level), and Cluster 3 (high NP expression in macrophages and intermediate secreted TNF-α level), we were able to discern specific molecular and cytokine response trends that shed light on how different patient groups may react to IAV exposure. In this context, Cluster 2 served as our reference subgroup.

Cluster 2, identified by its muted NP expression in macrophages during mock infections, likely represents individuals with limited previous exposure to IAVs. Their pronounced upswing in secreted cytokines TNF-α and IFN-γ upon H3N2 IAV exposure aligns with prior research (Miettinen et al., 2001; Seo and Webster, 2002), painting a picture of an immune system rallying its defenses against a relatively new viral threat.

In contrast, the pronounced NP expression in Cluster 3 during mock infections could signal a history of exposure to IAVs, possibly without overt clinical manifestations. This silent sensitization seems to have equipped them with a faster and more intense immune response upon subsequent H3N2 challenges (Gschoesser et al., 2002). The pronounced secretions of IL-2 and IFN-γ at the onset of H3N2 IAV exposure speak to this heightened immune vigilance.

Cluster 1, distinguished by its pronounced TNF-α secretion, unveils another facet of the immune response. With a demographic skew toward older individuals and those with a history of lung cancer, the heightened TNF-α secretion possibly hints at a backdrop of chronic inflammation, or even a predisposition toward malignant processes (Balkwill, 2006). This elevated TNF-α response, even in the absence of an active viral challenge, suggests underlying immune dysregulation. This observation, coupled with previous findings (Le Goffic et al., 2007), suggests that TLR3 overexpression on epithelial cells could be inducing IL-2, TNF-α, and IFN-γ upon IAV challenge. The amplified PD-L1 on macrophages, potentially facilitating viral proliferation (Ning et al., 2023), adds another layer to this narrative. When juxtaposed against Clusters 2 and 3, Cluster 1 exhibited a subdued cytokine response, possibly underpinning a hampered CD8^+^ T cell defense against IAV, especially in the older demographic (Liu et al., 2012). Taking together, our observations indicate that Cluster 1 individuals might be grappling with immunosenescence. This finding, in concert with the other cluster-specific observations, accentuates the multifaceted nature of the immune response to IAV and underscores the importance of individual patient characteristics in tailoring therapeutic strategies.

This study sheds light on the immune response and inflammasome activation during IAV infection using a human *ex vivo* model, but several caveats exist. First, we didn’t use a western blot for IAV NP in this study, but we had validated infections through quantitative real-time PCR of IAV RNA in our previous study (Huang et al., 2018). Elevated NP expressions typically indicate active IAV infection (Portela and Digard, 2002; Martinez-Colon et al., 2019). Second, prior research underscores the protective role of IFN-α against IAV in specific human tissues (Pothlichet et al., 2013; Matos et al., 2019). In light of existing evidence (Panda et al., 2010; McKendry et al., 2016; Galani et al., 2017) and our prior empirical observations (Huang et al., 2018; Hung et al., 2020), we strategically chose to employ flow cytometry for the identification of IAV NP, PD-L1, and TLR1-3 in this research. However, the implementation of Western blotting or quantitative PCR is indispensable for the accurate quantification of protein and mRNA levels of NP, PD-L1, and TLR1-3 in T-cells, macrophages, and epithelial cells, facilitating a more comprehensive understanding of host responses (Zhang Q. et al., 2020). We intend to incorporate these pivotal methodologies in subsequent studies to augment the depth and precision of our insights. Third, while our *ex vivo* model offers direct insights into human tissue responses, it inevitably lacks the systemic interplay present in *in vivo* conditions. This limitation means that while our findings provide foundational knowledge, *in vivo* studies will be essential for translating these observations into clinical practice. Fourth, our primary focus on CD3^+^ and CD8^+^ T cells may have missed the nuanced responses from other specialized T cell subsets. Including these subsets could help elucidate the full spectrum of T cell responses to IAV infection. Fifth, the relatively limited sample size and short incubation period may restrict the scope and generalizability of our findings. Future investigations employing larger sample sizes and prolonged incubation periods can offer more robust and detailed insights.

In conclusion, our study has illuminated the nuanced responses of human lung tissue to *ex vivo* H3N2 IAV infections, underscoring the central role of NP-expressing macrophages in the formative stages of the immune response. Aging, histories of lung cancer, and previous encounters with IAV emerge as pivotal factors that shape and modulate these responses. The molecular markers we’ve identified, notably NP expression in macrophages and TNF-α secretion during mock infections, offer a valuable schema to categorize patients based on the nature and magnitude of their immune reactions and subsequent inflammasome activations. Our *ex vivo* model stands out in its capability to mirror the myriad ways host factors mold innate immune responses to IAVs. This understanding, though preliminary, offers a bedrock upon which we can design more tailored and effective preventive and therapeutic interventions against H3N2 IAV and potentially other influenza viruses. To solidify our conclusions, studies with larger samples and a broader spectrum of immune cells are essential. This will enrich our understanding and improve strategies to manage IAV infections.

## Data availability statement

The original contributions presented in the study are included in the article/**Supplementary Material**. Further inquiries can be directed to the corresponding author.

## Ethics statement

The studies involving humans were approved by the Institutional Review Board at the Chang Gung Medical Foundation, Taoyuan, Taiwan. The studies were conducted in accordance with the local legislation and institutional requirements. The participants provided their written informed consent to participate in this study.

## Author contributions

C-GH: Conceptualization, Data curation, Formal Analysis, Funding acquisition, Investigation, Methodology, Resources, Software, Validation, Writing – original draft. Y-CW: Conceptualization, Data curation, Formal Analysis, Investigation, Methodology, Validation, Writing – original draft. M-JH: Data curation, Formal Analysis, Investigation, Methodology, Writing – review & editing. Y-JL: Data curation, Investigation, Methodology, Writing – review & editing. T-HH: Data curation, Investigation, Methodology, Writing – review & editing. P-WH: Data curation, Formal Analysis, Investigation, Methodology, Project administration, Validation, Visualization, Writing – original draft. S-LY: Data curation, Investigation, Methodology, Resources, Writing – review & editing. K-CT: Supervision, Writing – review & editing. S-RS: Supervision, Validation, Writing – review & editing. L-AL: Conceptualization, Data curation, Formal Analysis, Visualization, Writing – original draft.
