# Supplementary material for: Impact of patient characteristics on innate immune responses and inflammasome activation in ex vivo human lung tissues infected with influenza A virus
Source: Front Cell Infect Microbiol. 2023 Oct 13;13:1269329. doi: 10.3389/fcimb.2023.1269329 (PMC10611511; doi:10.3389/fcimb.2023.1269329)
Supplement: Supplementary file 1 [file Table_1.pdf]

Impact of Patient Characteristics on Innate Immune Responses and Inflammasome Activation in *Ex Vivo* Human Lung Tissues Infected with Influenza A Virus

Chung-Guei Huang[1,2,3], Yi-Cheng Wu[4,5,6], Ming-Ju Hsieh[4,5], Ya-Jhu Lin[1], Tzu-Hsuan Hsieh[1], Po-Wei Huang[1], Shu-Li Yang[1], Kuo-Chien Tsao[1,2,3], Shin-Ru Shih[1,2,3], Li-Ang Lee[5,6,7*]

Supplementary Table 1 Antibodies for flow cytometric analysis.

Molecular marker	Antibody	Catalog number	Clone	Manufacturer	Fluorochrome
CD3	mouse anti-human CD3	561806	UCHT1	Becton Dickinson Biosciences	FITC
CD4	mouse anti-human CD4	565994	SK3		APC
CD8	mouse anti-human CD8	560959	HIT8a		PE
CD45	mouse anti-human CD45	564105	HI30		PerCP-Cy™5.5
EpCAM	mouse anti-human CD326	565685	EBA-1		BV786
HLA-DR	mouse anti-human HLA-DR	561358	G46-6		APC-H7
PD-1	mouse anti-human CD279	562516	EH12.1		BV421
PD-L1	mouse anti-human CD274	563741	MIH1		APC
TLR1	mouse anti-human CD281	566430	GD2.F4		BV421
TLR2	mouse anti-human CD282	742767	11G7		BV510
TLR3	anti-human CD283	315009	40C1285.6	BioLegend, Inc.	PE
NP	Influenza A NP monoclonal antibody	MA1-7322	D67J	Thermo Fisher Scientific	FITC

Abbreviations: CD, cluster of differentiation; EpCAM, epithelial cell adhesion molecule; HLA-DR, human leukocyte antigen-DR; NP, nucleoprotein; PD-1, programmed death 1; PD-L1, programmed death-ligand 1; TLR, toll-like receptor.
